# Risk of metachronous colorectal cancer associated with polypectomy during endoscopic diagnosis of colorectal cancer

**DOI:** 10.1007/s00384-024-04722-8

**Published:** 2024-10-02

**Authors:** James Giulian Fiori, Steven Kim, Marina Helen Wallace, Samantha Rankin, Oyekoya Taiwo Ayonrinde

**Affiliations:** 1 Department of Gastroenterology and Hepatology, Fiona Stanley Hospital, 11 Robin Warren Drive, Murdoch, Perth, WA WA 6150 Australia; 2 Department of Colorectal Surgery, Fiona Stanley Hospital, Perth, WA Australia; 3 Medical School, The University of Western Australia, Perth, WA Australia; 4 Clinical Services, Fiona Stanley Hospital, Perth, WA Australia; 5 Medical School, Curtin University, Bentley, WA Australia

**Keywords:** Colorectal cancer, Polypectomy, Colonoscopy, Surveillance, Anastomosis

## Abstract

**Background and aim:**

There are conflicting reports regarding the risk of metachronous colorectal cancer (CRC) subsequent to colonoscopy with polypectomy or biopsy performed concurrently with diagnostic biopsies for CRC. We aimed to establish the 5-year risk of CRC in patients who had synchronous polypectomy or biopsies during the colonoscopy at which CRC was diagnosed.

**Methods:**

This is a single-centre retrospective case–control study of adults who underwent surgical resection for CRC over a 2-year period (January 2016 to December 2017). Colonoscopy details of interest were the location of the CRC, polypectomy and non-CRC biopsy sites. In patients with CRC at index colonoscopy, we sought associations between the occurrence of metachronous CRC and the sites from which endoscopic specimens had been obtained.

**Results:**

Our study population comprised 225 patients with a median (IQR) age of 71 (60–77) years. Polypectomy or biopsy at a non-CRC site had been performed during the index colonoscopy in 108 patients (48%), including 83 (37%) polypectomies outside the surgical resection field. There were 8 (3.6%) metachronous CRCs: 1 (0.4%) at the site of endoscopic mucosal resection for a 15-mm sessile serrated lesion, 3 (1.3%) anastomotic site CRCs and 4 (1.8%) at other sites within the colon. There was no significant difference in the prevalence of metachronous CRC in patients who underwent polypectomy/biopsy at the index colonoscopy compared with those who did not (1.9% vs. 5.1%, *p* = 0.283).

**Conclusion:**

There was no significant increased risk of metachronous CRC subsequent to synchronous polypectomy or biopsy during the colonoscopy at which CRC was diagnosed.

## Introduction

Up to 3% of patients who undergo surgical resection for colorectal cancer (CRC) develop metachronous CRC [1]. Whilst metachronous CRC can result from incomplete polypectomy or missed neoplastic lesions, a potentially avoidable and under-recognised cause may be tumour implantation when polypectomy, biopsy, tattooing or other endoscopic manipulation of the mucosa occurs during the index colonoscopy [2].

CRC cells are known to be exfoliated into the lumen of the colon [3] and can result in anastomotic recurrence [4], seeding to stapling devices [5], suture lines [6] and fistulae tracks [7]. Viable colorectal cancer cells have been demonstrated to be present in the working channel of colonoscopes after tumour biopsy [2]. As such, there is a theoretical risk of implanting tumour cells when performing polypectomy or other mucosal manipulation during the same colonoscopy at which a CRC is diagnosed [2]. Previous studies have demonstrated a small risk of tumour seeding in this context.

Backes et al. demonstrated a risk of up to 0.6% of tumour implantation when polypectomy or biopsy is performed during the index colonoscopy at CRC diagnosis [2]. That study identified identical molecular signatures between primary and metachronous tumours in cases where recurrence occurred at the site of previous polypectomy [2]. By contrast, Tan et al. did not demonstrate a significantly increased risk of tumour implantation after synchronous polypectomy [8]. Approximately 40% of colonoscopies where CRC is identified have synchronous polyps or other lesions present that may warrant polypectomy or biopsy [9]. Some endoscopists elect to not perform polypectomy or biopsy of these lesions due to a perceived risk of tumour cell implantation. This approach potentially exposes the patient to a further endoscopic procedure and a small risk of high-risk polyps becoming malignant whilst awaiting the first surveillance colonoscopy post-CRC resection.

Australian practice is likely to be varied, as there is a paucity of guidelines to inform best practice regarding the management of such lesions. Therefore, our study aimed to investigate the risk of metachronous CRC following polypectomy or biopsy of synchronous lesions concurrently identified during colonoscopy at which CRC was diagnosed in an Australian tertiary hospital network.

## Method

### Study population

This is a single-centre retrospective cohort study including adults (age ≥ 18 years) who underwent bowel resection for colorectal cancer from the 5th of January 2016 to 21st of December 2017, as this fulfilled 5 years of follow-up. Participants were excluded if they had a prior diagnosis of Lynch syndrome; familial adenomatous polyposis (FAP); MUTYH-associated polyposis (MAP); serrated polyposis syndrome (SPS); ulcerative colitis extending proximal to the sigmoid colon with a diagnosis for > 8 years; or Crohn’s disease involving > 1/3 of colon or complicated anorectal disease, with a diagnosis for > 8 years before bowel resection. Participants were also excluded if there was CRC but no index colonoscopy report available on the medical record or if there was no available follow-up data to enable identification of recurrences within 5 years of surgery. Resections performed for anal squamous cell carcinoma, carcinoid tumours and colovesical fistulae were also excluded. Participants were not excluded if they died prior to 5 years post-surgery.

### Data collection and extraction

Reports from the colonoscopy at which CRC was first diagnosed, considered the index colonoscopy, were reviewed to obtain data regarding polypectomy/biopsy location, size and method. Operation reports were reviewed to identify the type of surgery performed and determine whether the polypectomy/biopsy was outside the surgical margins. Clinical records (clinic letters, colonoscopy reports, discharge summaries) up to 5 years post-surgery were reviewed to identify cases of metachronous CRC, defined as recurrence occurring between 6 months and 5 years post resection. The location of metachronous lesions was recorded and compared with the resection surgery and site of polypectomy/biopsy. If a metachronous CRC occurred at the same location of polypectomy/biopsy during the index colonoscopy, this was deemed to be possibly due to tumour seeding. Other demographic and medical data collected included age at CRC diagnosis, medical history and complications during index colonoscopy (defined as either a colonic perforation or bleed that required a repeat colonoscopy within 3 weeks). The rate of metachronous CRC within 5 years post-tumour resection was calculated, and the percentage of cases potentially attributable to tumour seeding was determined.

### Statistical analysis

Statistical analysis was performed using Jamovi software (Version 2.3.21.0). Descriptive data was presented as median (IQR) or percentages. The proportion of metachronous CRC in the group of patients who underwent polypectomy or biopsy at the index colonoscopy was compared to the group who did not undergo polypectomy or biopsy. The proportion of metachronous CRC in those who underwent polypectomy or biopsy at index colonoscopy outside the surgical resection margins was compared to those who underwent polypectomy or biopsy at index colonoscopy inside the surgical resection margins. The rate of metachronous CRC at anastomotic sites was compared to those at polypectomy/biopsy sites. The median time between surgery and metachronous CRC diagnosis was compared between polypectomy site and anastomotic site recurrences. Comparisons were performed using Fisher’s exact test, Chi-square test and Kruskal–Wallis *H*-test as appropriate.

### Ethical considerations

Ethics approval was waived by the site Quality and Safety Assurance Unit, as this was a low-risk retrospective data analysis with de-identified data which does not result in any change in index patient management, thereby qualifying as a quality improvement project: Governance, Evidence, Knowledge and Outcome (GEKO) Quality Activity Number 50087.

## Results

### Demographic and clinical data

**Fig. 1:**
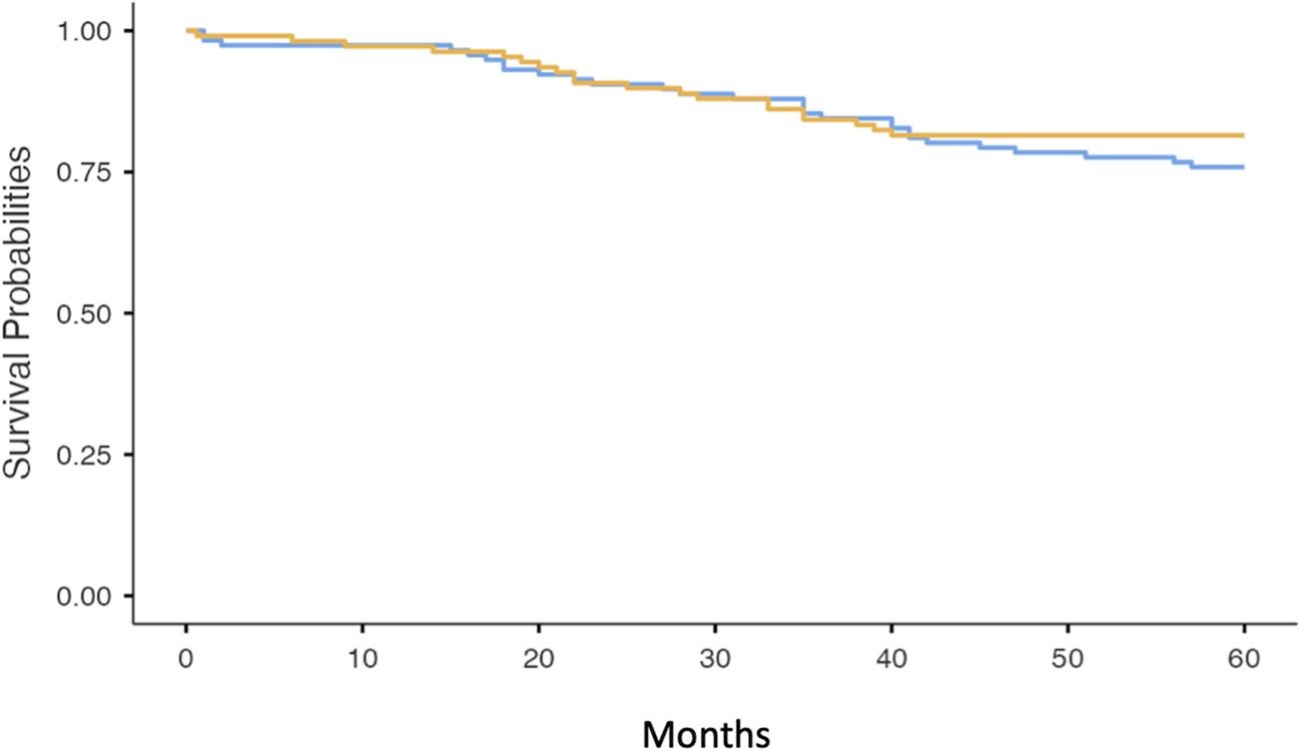
Kaplan-Meir survival curve of all-cause mortality in patients who underwent polypectomy/biopsy of synchronous lesions at index colonoscopy (orange line) compared to those who did not undergo polypectomy/biopsy (blue line)

**Fig. 2:**
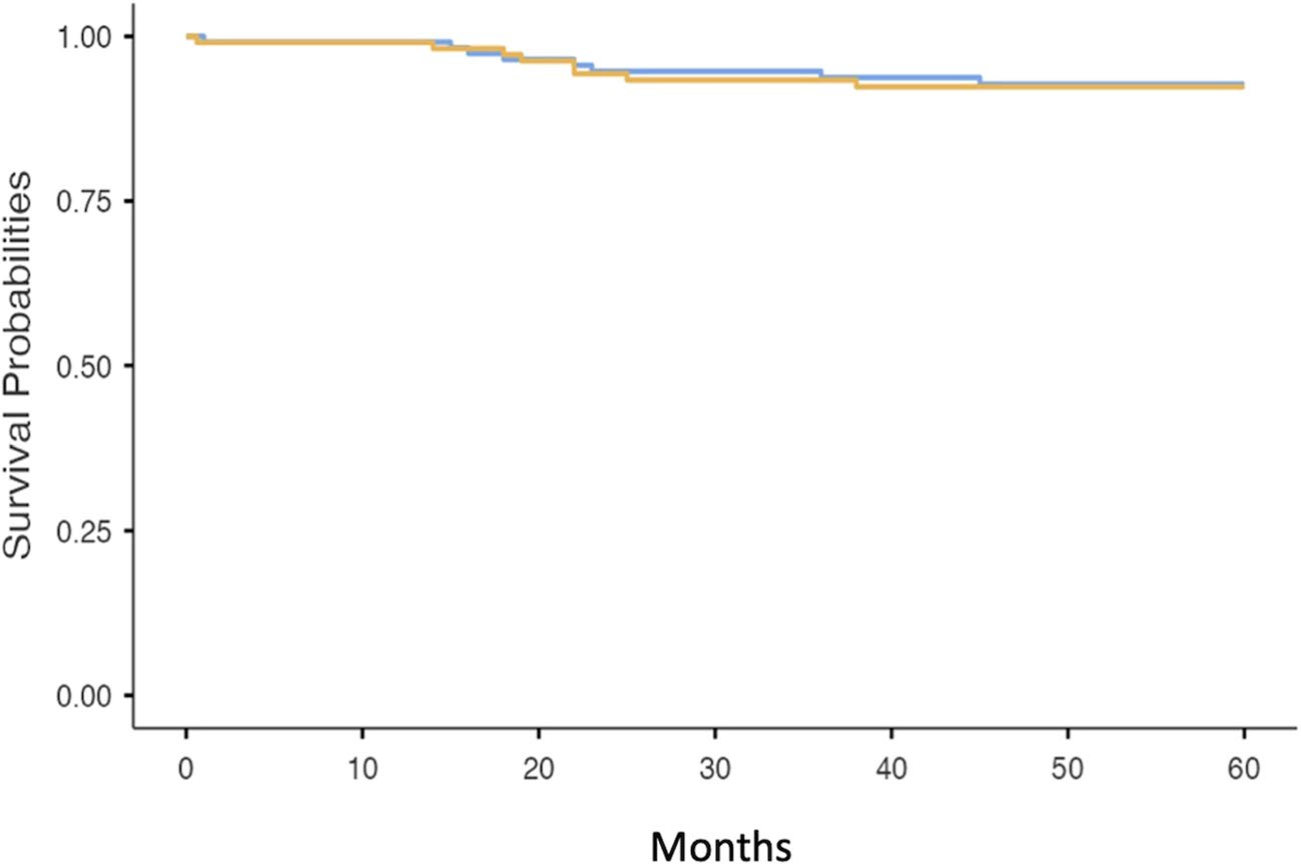
Kaplan-Meir curve of colorectal cancer-related death in patients who underwent polypectomy/biopsy of the synchronous lesion at index colonoscopy (orange line) compared to those who did not undergo polypectomy/biopsy (blue line)

Two hundred and ninety-four patients underwent surgical resection of CRC during the study period. After exclusions, a total of 225 patients were included in the study. Index colonoscopies were performed for varied indications that included symptoms, screening and surveillance and not an exclusive CRC screening program. Twenty-two patients were excluded as there was no colonoscopy record available, 20 were not resections for CRC but were actually other types of surgeries such as reversal of loop ileostomy or colostomy following previous CRC surgery, and 10 were lost to follow-up. Reasons for exclusion are outlined in Table 1. The median (IQR) age was 71 (60–77) years. The most common sites of primary CRC were the rectum, sigmoid colon and caecum; however, 161 were at or distal to the splenic flexure (Table 2). One patient had a complication from the index colonoscopy, which was a post-polypectomy bleed that required endoscopic therapy. As outlined in Fig. 1 and Fig. 2, 48 patients (21%) died within a period of 5 years post-surgery, 16 of which were CRC-related deaths.

**Table 1:** Reasons for exclusion from the data set

Reason for exclusion	Number
Reversal of ileostomy	1
Not a resection (tumour adherent to the abdominal wall)	1
Lost to follow-up	10
Loop ileostomy	7
Jejunal tumour found on exploratory laparotomy (no preceding colonoscopy)	1
Lynch syndrome	4
Loop colostomy	6
EUA not for CRC	1
Colonoscopy report not available	22
Reversal of ileostomy	1
Unable to access record	1
Repeat patient in data set	1
Ulcerative colitis	1
Cervical cancer adherent to rectum	1
Colovesical fistula surgery	1
Serrated polyposis syndrome	1
Carcinoid tumour	3
Resection for scar from radiotherapy for anal SCC	1
Anal SCC	5

**Table 2:** Location of primary colorectal cancers resected during the study period

Cancer location	Primary CRC site
Appendix	2
Ascending colon	17
Caecum	22
Caecum and ascending colon	1
Caecum and lateral abdominal wall	1
Caecum and rectum	1
Caecum and transverse colon	1
Descending colon	4
Distal transverse colon	1
Hepatic flexure	5
Hepatic flexure and ascending colon	1
Ileocolonic anastomosis	1
Ileum	2
Left colon	1
Rectosigmoid junction	6
Rectum	89
Rectum and caecum	2
Sigmoid colon	53
Splenic flexure	4
Splenic flexure and ascending colon	1
Transverse colon	10

### Rate of metachronous colorectal cancer and tumour seeding

One hundred and eight patients had a polypectomy or biopsy (at a site other than the CRC site) performed during the index colonoscopy. Eighty-three patients had a polypectomy/biopsy performed outside the margins of the surgical resection, with 25 performed within the margins of the surgical resection.

There were eight (3.56%) cases of metachronous CRC. One (0.44%) occurred at the site of previous polypectomy. This was a large (15 mm) sessile serrated lesion without dysplasia, resected with hot snare endoscopic mucosal resection (EMR). It was unclear whether it was resected en bloc or piecemeal. The patient underwent further surveillance colonoscopy at 6 months and then 12 monthly in line with national surveillance recommendations [10]. Three (1.33%) metachronous CRCs occurred at anastomotic sites, and four (1.78%) occurred at other sites within the colon. There was no significant difference between the recurrence rate at anastomotic sites, previous polypectomy/biopsy sites and other sites within the colon, *p* = 0.417 (Table 3).

**Table 3:** Total number of patients post exclusion, and number of recurrences in each subgroup

	Number (%)
N (post exclusion)	225
Recurrence of colorectal cancer	8 (3.56%)
Recurrence of CRC at sites of previous polypectomy or biopsy	1 (0.44%)
Recurrences of CRC at anastomosis	3 (1.33%)
Recurrences of CRC not at the site of prior polypectomy or biopsy	4 (1.78%)

**Fig. 3:**
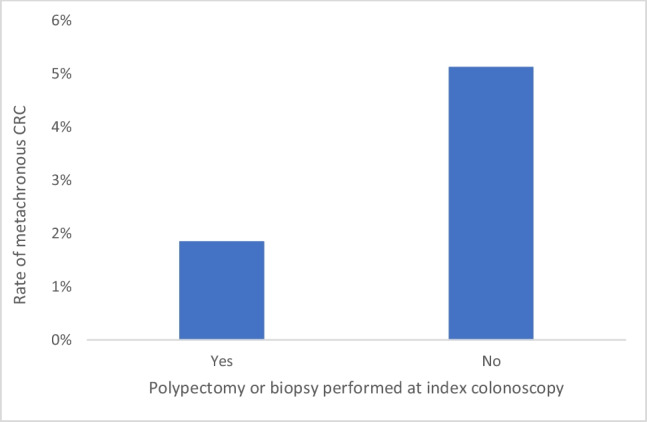
Rate of metachronous CRC in patients who underwent polypectomy or biopsy at the index colonoscopy vs. those who did not. This was not statistically significantly different (*p* = 0.283)

There were two cases (1.85%) of metachronous CRC in patients who underwent polypectomy/biopsy at the index colonoscopy. In comparison, the group that did not undergo polypectomy/biopsy during index colonoscopy had six (5.13%) cases of metachronous CRC (Fig. 3). The difference between those who had polypectomy/biopsy compared to those who did not was not statistically significant, *p* = *0.283.*

The median time from surgery to metachronous CRC was 2.16 years. Only 50% of patients who had a recurrence were up to date with surveillance colonoscopy (Table 4).

**Table 4:** Median time from CRC diagnosis and surgery to metachronous CRC diagnosis and first surveillance colonoscopy

	Median time from CRC diagnosis to metachronous CRC diagnosis	Median time from surgery to metachronous CRC diagnosis	Median time from CRC diagnosis to first surveillance colonoscopy	Median time from surgery to first surveillance colonoscopy	% up to date with surveillance colonoscopy at time of recurrence
Overall	2.25 (1.64–4.04) years	2.16 (1.44–3.86) years	1.37 (0.97–1.83) years	1.29 (0.65–1.72) years	50%
Anastomotic recurrence	1.78 (1.32–2.25) years	1.62 (1.16–2.15) years	1.00 (0.94–1.39) years	0.96 (0.82–1.29) years	66.6%
Polypectomy site recurrence	5.01 years	4.86 years	0.44 years	0.29 years	100%
Other site recurrence	2.75 (1.64–4.04) years	2.60 (1.47–3.86) years	1.85 (1.55–2.42) years	1.79 (1.38–2.33) years	25%

As outlined in Table 5 the majority of (five out of eight) cases of recurrence arose from original tumours in the left side of the colon, and the majority of recurrences (five out of eight) occurred in patients who underwent laparoscopic resections rather than open resections. Specific indications for the index colonoscopy, histological features and locations of the original tumour and recurrent tumour are outlined in Table 6.

**Table 5:** Location of CRC recurrences and surgical characteristics

Recurrence number	Gender	Site of the original tumour	Polypectomy site or anastomotic site recurrence	Open vs. laparoscopic surgery	Previous abdominal or pelvic surgery
1	Female	Sigmoid	Polypectomy site recurrence	Open	No
2	Male	Rectum	Anastomotic	Open	No
3	Female	Sigmoid	Anastomotic	Laparoscopic	No
4	Male	Hepatic flexure	Anastomotic	Open	Yes
5	Female	Rectum	No	Laparoscopic	No
6	Female	Ascending	No	Laparoscopic	Yes
7	Female	Caecum	No	Laparoscopic	No
8	Male	Sigmoid	No	Laparoscopic	No

**Table 6:** Features of CRC recurrences- Type of recurrence, indications for index colonoscopy, histological characteristics and time between index colonoscopy and recurrence

Patient	Type of recurrence	Indication for index colonoscopy	Histological features of polypectomy (if applicable)	Histological features of original CRC	Histological features of recurrent CRC	Time between index colonoscopy and recurrence
1	Polypectomy site	Rectal bleeding, constipation	Sessile serrated adenoma/polyp with no traditional dysplasia from transverse colon. Multiple tan polypoid fragments, ranging in size from 2 up to 15 × 5 × 4 mm	Low-grade *sigmoid* adenocarcinoma, 3/18 positive nodes, pT3 N1 M0.	*Transverse colon* high-grade adenocarcinoma, 0/14 positive nodes, pT2 N0 Mx.	4.9 years
2	Anastomotic site	Incidental rectal mass on CT	NA	Low-grade adenocarcinoma arising in high-grade tubulovillous adenoma from the *rectum* 0/10 positive nodes, pT1 N0 MX.	*Anastomosis* biopsy showed focal recurrence of adenocarcinoma.	1.8 years
3	Anastomotic site	Positive FOBT on National Bowel Cancer Screening Program	NA	Low-grade *sigmoid* adenocarcinoma of usual intestinal type with mucinous differentiation; 5 of 12 lymph nodes positive, pT4a N2a MX.	*Anastomosis* biopsy showed fragments of colonic mucosa with a moderately differentiated adenocarcinoma composed of complex glandular structures surrounded by desmoplastic stroma. No lymphovascular space invasion identified.	0.88 years
4	Other sites	Weight loss and abdominal pain	NA	Low-grade adenocarcinoma of *hepatic flexure* (moderately differentiated), invades muscularis propria (pT2), 0/14 positive nodes.	Low-grade invasive colonic adenocarcinoma at *sigmoid.* The invasive adenocarcinoma may be arising on a background of a sessile serrated polyp (sessile serrated adenoma) with low- and high-grade dysplasia.	1.73 years
5	Anastomotic site (was a second rectosigmoid recurrence)	Surveillance following a previous CRC resection	NA	Low-grade adenocarcinoma of intestinal type, *rectum*, 4/7 positive nodes, pT4a N2a MX.	*Anastomosis* biopsy showed invasive adenocarcinoma; The tumour comprises infiltrating malignant glands lined by pleomorphic malignant cells set in desmoplastic stroma.	2.72 years
6	Other sites	Iron deficiency anaemia	NA	Low-grade mucinous adenocarcinoma, *ascending colon*, 0/21 positive nodes, pT3 N0 MX.	Flat sessile plaque-like tumour of *sigmoid.*	3.78 years
7	Other sites	Diarrhoea and rectal bleeding	NA	Low-grade adenocarcinoma, *Caecum*, 11/15 positive nodes, pT4a N2b MX.	Metastatic adenocarcinoma of *rectum*, with morphology and immunophenotype in keeping with the known mucinous colorectal adenocarcinoma.	4.82 years
8	Other sites	Incidentally found sigmoid mass on MRI of liver lesion	NA	Adenocarcinoma of usual intestinal type of *sigmoid*. 0/15 positive nodes, pT2 pN0 pMX.	High-grade adenocarcinoma of *hepatic flexure*, 4/7 positive nodes, pT4a pN2a MX.	1.34 years

## Discussion

In this study, we found a low risk of metachronous CRC when polypectomy/biopsy was performed during the index colonoscopy, with the risk of recurrence not significantly different compared to the group of patients who did not have polypectomy/biopsy performed. There was only one case of metachronous CRC at the same site of a polypectomy. It should be noted this polyp was a large (15 mm), sessile serrated adenoma without dysplasia resected with hot EMR; however, it was unclear whether it was resected en bloc or piecemeal; hence, it is difficult to determine whether the metachronous CRC may have been due to incomplete resection rather than tumour seeding.

### In context with published literature

Tan et al. [8] found a similar metachronous CRC rate of 2.8% and found a 1.4% rate at the site of previous polypectomy. They also found no significant difference in metachronous CRC between patients who had polypectomy within the surgical margins compared to those who had polypectomy outside the surgical margins, suggesting that the risk of tumour implantation is not significant. An Australian study in 2010 found a rate of metachronous CRC of 2.5% and no cases of recurrence at sites of previous polypectomy [11]. In our study, we found a 0.44% prevalence of metachronous CRC in the same location of the bowel where a previous polyp was resected. Although Backes et al. [2] reported the risk of tumour seeding during colonoscopy during which CRC is diagnosed to be 0.3–0.6%, we are unable to attribute the metachronous CRCs in our study to tumour seeding as we did not collect data on specific tumour biology to confirm this. Our finding of an anastomotic recurrence rate of 1.33% is similar to the cumulative incidence of 2.7% described in a recent meta-analysis that identified the risk of anastomotic recurrence to be highest in the first 36 months post-CRC resection, subsequently decreasing [12].

A notable finding is the similarity in anastomotic and polypectomy site metachronous CRC rate, which may potentially reflect seeding during surgical manipulation of the CRC. Both anastomotic recurrences and polypectomy site recurrences represent mucosal breaches that pose a risk of tumour cells that are exfoliated into the lumen of the bowel being implanted into the mucosa. Whilst some cases of anastomotic recurrence may be explained by incomplete resection margins of the tumour [13], exfoliated tumour cells shed into the lumen becoming implanted into the anastomotic site are a well-recognised cause [14–16]. Between 40 and 70% of exfoliated colorectal cancer cells are viable [16, 17], with viable tumour cells detected at anastomotic sites [16] and found within surgical stapler devices [18]. Endoscopic submucosal dissection has also been shown to carry a risk of tumour cell implantation where the post-dissection ulcer provides a surface for viable cancer cells to implant [19]. Our findings of a similar rate of metachronous CRC at polypectomy sites compared to anastomotic sites suggest a potentially similar risk.

Anastomotic cancers in our study occurred predominantly but not exclusively in the left side of the colon (two out of three). The risk of anastomotic site implantation is thought to be particularly higher for rectal cancers, where more manipulation of the tumour is required in the narrow pelvis [20], the anastomotic site is generally closer to the tumour, and more exfoliated cells are able to spread within the lumen at this site [21]. This risk was recognised as early as the 1940s [22] with the introduction of rectal stump washout to reduce the risk of tumour cell implantation, with it now being common practice in 60–87% of surgeons in the UK and USA [23–25] during anterior resection for colorectal cancer. Irrigation is less frequently performed in resections of tumours in more proximal areas of the colon due to a lower risk of anastomotic recurrence at these sites [26]. This raises the idea of whether irrigation prior to polypectomy would reduce the risk of tumour implantation at polypectomy sites, as this is not currently routine practice.

Another notable finding was that only 50% of cases of metachronous CRC were up to date with their surveillance colonoscopies at the time of diagnosis. This highlights the need for prioritisation of appropriate timing of high-quality post-CRC surveillance colonoscopy, with a previous study demonstrating that 43.0% of metachronous CRC are attributable to missed lesions or non-adherence to surveillance advice [27]. It should be noted that we did not collect data on the quality of colonoscopies given the retrospective nature of the study and the lack of consistent reporting of the quality of the colonoscopy, which is also a significant risk factor for metachronous CRC by increasing the possibility of missed lesions [27]. The predominant sites of detected metachronous CRC are similar to sites associated with missed lesions during colonoscopy [28], underscoring the additional importance of quality surveillance colonoscopy [29].

### Strength and limitations

A strength of this study is that it provides local Australian data, and possibly reassurance, regarding a low risk of metachronous CRC associated with synchronous polypectomy during colonoscopy in which CRC is biopsied. The study was performed at a single centre, which therefore assumes similar clinical practices with regard to biopsies, tattooing and snare polypectomy techniques and in regards to surveillance schedule. A further strength of this study is that it evaluated real clinical practice, compared to some previous studies which assessed the risk of tumour implantation in this context using in vitro models [30].

A limitation of this study is its single-centre retrospective cohort design. A further limitation was the fact that 69 patients (23.5%) of the original 294 patients were excluded. A notable limitation of our study was that we were unable to determine whether polypectomies/biopsies were done prior to or after obtaining a biopsy of the CRC, due to the absence of this data. Nevertheless, it is routine practice to perform polypectomy for synchronous lesions at the institution this study was conducted at. This is an important consideration if a future prospective study was to be performed, as a mucosal breach by performing polypectomy or biopsy after obtaining a biopsy of the CRC could pose a higher risk of tumour implantation. This risk could be further increased by using the same biopsy forceps used for CRC biopsy to thereafter take other colorectal biopsies. Furthermore, our study did not compare molecular biomarkers of the case of metachronous CRC with the original CRC to confirm it was indeed a case of tumour implantation as opposed to being from a residual polyp.

Our findings carry important clinical implications. Firstly, given the risk of metachronous CRC at the same site of polypectomy appears to be low, performing polypectomy during the index colonoscopy at CRC diagnosis may be safe and avoid the need for repeated colonoscopies prior to or shortly after surgery. In Australia, routine surveillance colonoscopy is performed 12 months after surgical resection of colorectal cancer [10]. Thus, removing high-risk polyps at the time of index endoscopy would avoid the need for a potentially unnecessary colonoscopy sooner than this and hence avoid the risks and costs associated with a further procedure. Also, polypectomy at index colonoscopy minimises delays to surgery, as in some cases a repeat colonoscopy would be required to resect high-risk polyps prior to surgery.

In conclusion, in our study of an Australian cohort, we did not demonstrate a significant increase in metachronous CRC following polypectomy or biopsy of synchronous lesions during colonoscopy at which CRC was diagnosed. Despite the theoretical concern, the polypectomy group did not have a higher occurrence of metachronous CRC. Thus, it may not be inappropriate for high-risk polyps outside the expected CRC resection field to be resected during the colonoscopy at which CRC is diagnosed. Our findings also underscore the importance of appropriately timed high-quality surveillance colonoscopy after resection of CRC.

## Data Availability

The data that support the findings of this study are not openly available due to reasons of sensitivity and are available from the corresponding author upon reasonable request.
